# Galena Medical Society

**Published:** 1857-06

**Authors:** H. Newhall, W. S. Barker

**Affiliations:** President; Secretary


*Galena Medical Society.*

Pursuant to a call, signed by the physicians of this city, a meeting was held at the office of Drs. Newhall and Hempstead, on Thursday evening, April 2d, for the purpose of organizing a City Medical Society.

Dr. Newhall was called to the chair, and Dr. Barker appointed secretary.

A Constitution and By-Laws for the government of the society were presented, and unanimously adopted.

The following gentlemen were then elected officers for the ensuing year:—

Dr. H. Newhall, *President.*
Dr. J. S. Crawford, *Vice-President.*
Dr. W. S. Barker, *Secretary.*
Dr. E. D. Kittoe, *Treasurer.*

A list of charges for professional services were then presented, which, after thorough discussion, and some emendation, was unanimously adopted.

It was decided that the National Code of Ethics be adopted by the Society, and that its rules should strictly regulate the professional conduct of members in all cases.

The Society appointed a Delegate to the American Medical Association, which convenes at Nashville, Tenn., in May.

On motion of Dr. McKenney, it was resolved that the Fee

Bill be printed, with the names of the members of the Society and its officers appended thereunto.

The Secretary was required to furnish the editors of the city papers with an account of the proceedings of the meeting, requesting their publication.

The Society then adjourned to meet in quarterly session on the second Saturday in July next, at 7½ o'clock, P.M.

H. NEWHALL, M.D., *President.*

W. S. BARKER, M.D., *Secretary.*

